# The Role of Herpes Simplex Virus Type 1 Infection in Demyelination of the Central Nervous System

**DOI:** 10.3390/ijms21145026

**Published:** 2020-07-16

**Authors:** Raquel Bello-Morales, Sabina Andreu, José Antonio López-Guerrero

**Affiliations:** 1 Departamento de Biología Molecular, Universidad Autónoma de Madrid, Cantoblanco, 28049 Madrid, Spain; sabina.andreu@estudiante.uam.es (S.A.); ja.lopez@uam.es (J.A.L.-G.); 2 Centro de Biología Molecular Severo Ochoa, CSIC-UAM, Cantoblanco, 28049 Madrid, Spain

**Keywords:** HSV-1, oligodendrocytes, central nervous system, peripheral nervous system, demyelination, endogenous retroviruses, molecular mimicry

## Abstract

Herpes simplex type 1 (HSV-1) is a neurotropic virus that infects the peripheral and central nervous systems. After primary infection in epithelial cells, HSV-1 spreads retrogradely to the peripheral nervous system (PNS), where it establishes a latent infection in the trigeminal ganglia (TG). The virus can reactivate from the latent state, traveling anterogradely along the axon and replicating in the local surrounding tissue. Occasionally, HSV-1 may spread trans-synaptically from the TG to the brainstem, from where it may disseminate to higher areas of the central nervous system (CNS). It is not completely understood how HSV-1 reaches the CNS, although the most accepted idea is retrograde transport through the trigeminal or olfactory tracts. Once in the CNS, HSV-1 may induce demyelination, either as a direct trigger or as a risk factor, modulating processes such as remyelination, regulation of endogenous retroviruses, or molecular mimicry. In this review, we describe the current knowledge about the involvement of HSV-1 in demyelination, describing the pathways used by this herpesvirus to spread throughout the CNS and discussing the data that suggest its implication in demyelinating processes.

## 1. Introduction

Several neurotropic viruses may reach and infect the central nervous system (CNS) [1,2,3,4], including herpesviruses (herpes simplex virus type 1 (HSV-1), HSV-2, human cytomegalovirus (HCMV), and varicella zoster virus (VZV)), several arboviruses (West Nile, Japanese encephalitis, and chikungunya viruses), enteroviruses, henipaviruses, Ebola virus, and rabies virus [5]. These pathogens can cause a variety of nervous system diseases, such as encephalitis, flaccid paralysis, inflammatory immune disorders, or meningitis. Regarding the aetiology of demyelinating diseases (i.e., multiple sclerosis (MS)), several infectious agents, including viruses, bacteria, and protists, have been associated [6,7,8,9], in particular many viruses from the family Herpesviridae [10,11,12,13]. Epstein–Barr virus (EBV), human herpesvirus 6 (HH6), and HSV-1 have been linked to demyelinating diseases, although their role in these pathologies, and particularly in MS, is difficult to determine given their almost ubiquitous nature [11]. HSV-1 has also been involved in neurodegenerative disorders of the CNS [14,15,16,17].

It is not fully understood how HSV-1 reaches the CNS, although the most feasible explanation is retrograde transport through the olfactory or trigeminal tracts. It is also unknown whether herpes simplex encephalitis (HSE) is caused by the reactivation of the latent virus or primary infection, as both seem to be possible. Nevertheless, the poor correlation of HSE with primary infection suggests that HSE is more likely due to viral reactivation than to primo-infections [18]. However, latent HSV-1 has been demonstrated within several structures of the CNS, and the effects of infection with this virus in oligodendrocytes (OLs), the myelin-forming cells of the CNS, has also been reported.

In this review, we will describe the current knowledge about the involvement of HSV-1 in demyelination, discussing the pathways used by this herpesvirus to reach the CNS and the evidence implicating it in damage to OLs.

## 2. Herpes Simplex Type 1

HSV-1 is a double-stranded DNA herpesvirus belonging to the Alphaherpesvirinae subfamily [19]. It is an important neurotropic human pathogen that can infect also other species, especially non-human primates [20], as well as numerous cell types in vitro, although humans are the natural hosts [21]. HSV-1 is one of the most widely spread human viral pathogen, and around 67% of the global population have antibodies to this pathogen [22]. Primary infection takes place in epithelial cells and the virus is transmitted to new hosts via saliva. In this stage, HSV-1 typically causes labial and oral lesions, and although it may also cause genital herpes, the most common sexually transmitted type is herpes simplex virus type 2 (HSV-2) [23,24,25]. In addition, HSV-1 can cause severe pathologies such as encephalitis or keratoconjunctivitis [26]. HSE includes severe brain damage with hemorrhage, edema, and necrosis, and mostly affects the frontal and temporal lobes and the limbic system. It is generally considered that HSV-1 primary infections utilize oral routes of entry, given the common presentation of oral lesions. However, it has been argued that the acute oral lesions of human HSV-1 infections do not necessarily reflect oral host entry, and that the routes used for primary infection and reactivation are not necessarily the same [18].

After infection of epithelial cells, HSV-1 spreads to the peripheral nervous system (PNS), entering sensory neurons by fusion with the plasma membranes of their sensory terminals. Then, HSV-1 travels retrogradely to the cell body and establishes a latent infection in the trigeminal ganglia (TG) [27]. However, latent virus may also locate to CNS structures such as the olfactory bulb (OB), brainstem, or temporal cortex. During latency, the virus persists in the cell nucleus as an episome; the expression of lytic genes is repressed, and conversely the expression of latency-associated transcripts (LAT) begins. Periodically, HSV-1 may leave the latent state and reactivate, either spontaneously or in response to stimulation from immunosuppression, fever, ultraviolet light exposure, or injury to the tissues innervated by latently-infected neurons [19]. This process may lead to a recurrent lesion, but it may also proceed asymptomatically. Although reactivation is a complex process triggered by causes that are not fully understood, it has been demonstrated that the immune system plays a critical role, and in this regard host stress may lead to HSV-1 reactivation by increasing regulatory T cell (Treg) control of CD8+ T lymphocytes [28]. The roles of Tregs in the context of viral infections seem to be highly complex; Tregs may exert radically different roles depending on the infectious agent, the disease phase, or the genetic profile of the host, both suppressing antiviral immune responses and contributing to viral spread and establishment of latency, or conversely contributing to virus control [29]. Latency is also an epigenetically controlled process [19] in which changes induced by different stressors may trigger viral reactivation [30,31]. During reactivation, the virus travels anterogradely along the axon, replicating in the tissue of the dermatome innervated by the sensory neuron in which the virus established latency.

## 3. HSV-1 Infection of the CNS

HSV-1 may enter the CNS by two main routes: peripheral neurons and the bloodstream. Two cellular barriers (the blood–brain barrier and the blood–cerebrospinal fluid barrier) protect the CNS, separating it from the circulatory system [5,32]. However, other pathways to the CNS are available to pathogens, such as the olfactory system and the trigeminal nerve, which bypass the cellular barriers and provide a direct portal into the brain. Therefore, the trigeminal and olfactory nerves constitute direct routes to the brain that can evade the barriers imposed by the circulatory system [5].

**Figure 1:**
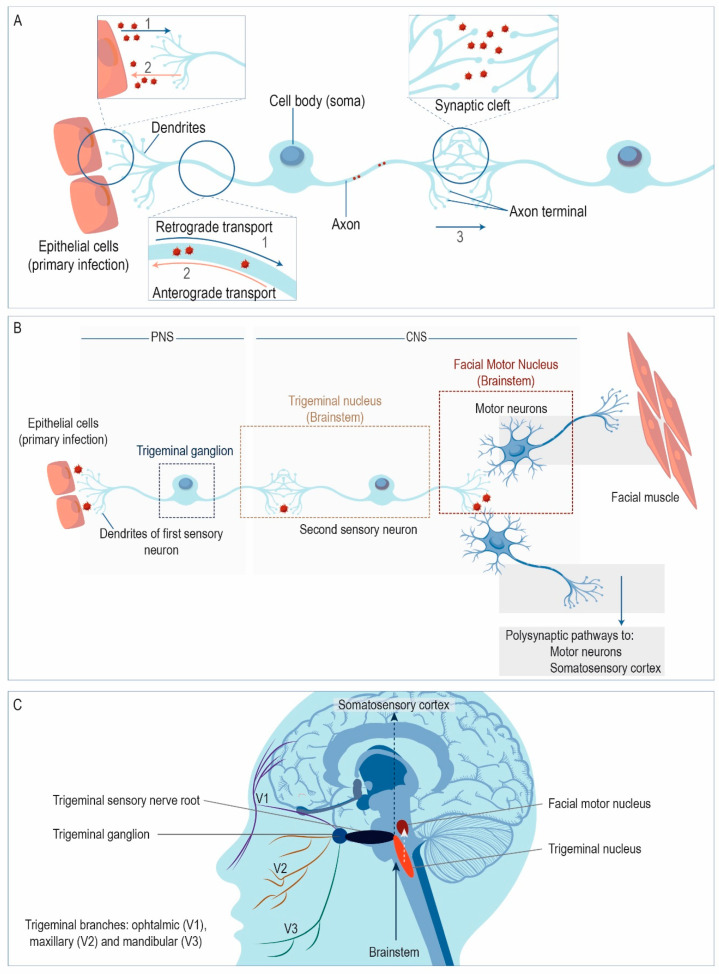
Spread of HSV-1 to the CNS via the trigeminal nerve. (**A**) HSV-1 may pass from the epithelia to the peripheral nervous system (PNS) by cell-to-cell spread between epithelial cells and nerve endings of sensory neurons that innervate them (1). The virus travels along the axon by retrograde transport to the cell soma of the sensory neuron, located in the trigeminal ganglion (TG). Conversely, HSV-1 can travel back by anterograde transport to the epithelial cells where the primary infection took place (2). The virus can also spread trans-synaptically, crossing the synaptic cleft (3). (**B**) After infection of epithelial cells, HSV-1 spreads to the PNS, entering sensory neurons by fusion with the plasma membrane of its nerve terminals. Then, HSV-1 travels retrogradely to the cell body and establishes a latent infection in the TG. Afterwards, the virus may enter the central nervous system (CNS) if it spreads trans-synaptically to the brainstem, from where it might spread to higher brain areas. (**C**) Spread to the CNS may take place through the three branches of the trigeminal nerve: ophthalmic, maxillary and mandibular. From here, the virus can access the trigeminal nucleus and other brain structures. (Structures are schematically represented and they are not drawn to scale).

**Figure 2:**
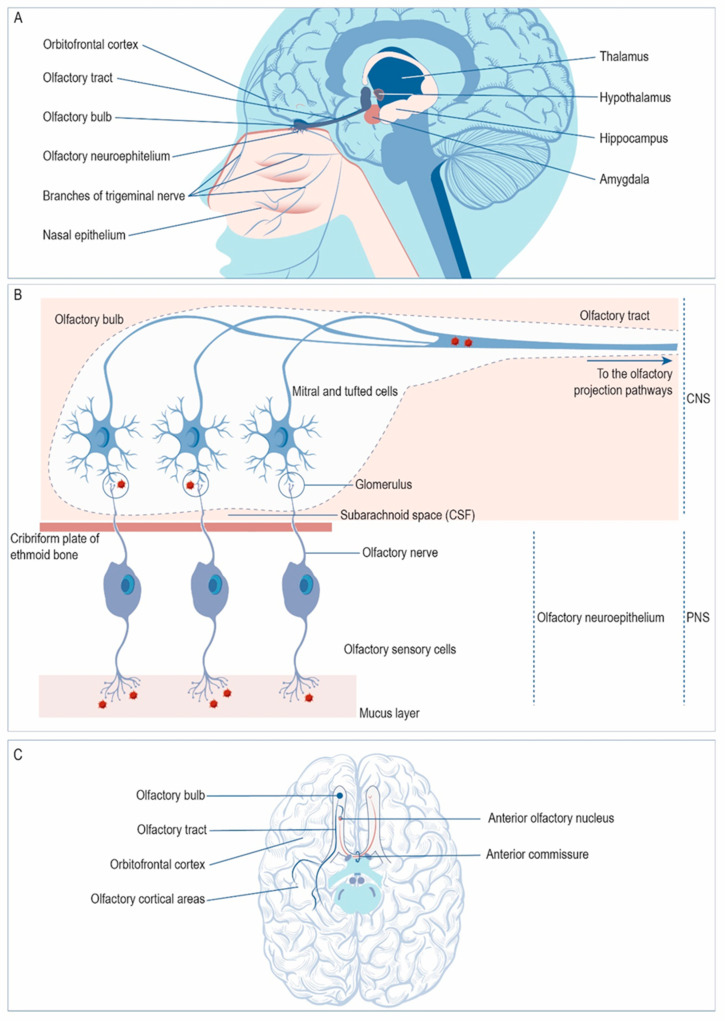
Spread of herpes simplex virus type 1 (HSV-1) to the central nervous system (CNS) via the olfactory nerve. (**A**) HSV-1 may enter the CNS via the olfactory neuroepithelium. From there, the virus may reach the olfactory bulb and then spread through the olfactory tract to reach limbic structures, such as the hippocampus, amygdala, or orbitofrontal cortex. (**B**) HSV-1 may reach the olfactory bulb infecting olfactory sensory cells, whose axons cross the ethmoid bone through the cribriform plate. These neurons form synapses with mitral and tufted cells in the glomeruli. The virus may infect these cells trans-synaptically at the glomeruli and spread towards the olfactory projection pathways. (**C**) Once in the olfactory tract, the virus may access the ipsilateral projection areas, such as the orbitofrontal cortex, via the lateral olfactory stria (in blue), or they may reach the contralateral olfactory structures through the anterior commissure via the medial olfactory stria (in red). (Structures are schematically represented and are not drawn to scale).

The neurotropic character of HSV-1 has been known for almost a century, since experimental corneal infection produced encephalitis in rabbits, suggesting that the virus propagated through axons and synapses by invasive proliferation [33]. It is currently assumed that primary HSV-1 infection takes place in epithelial cells and subsequently reaches the PNS by direct cell-to-cell retrograde spread to the nerve endings of nearby sensory neurons (Figure 1A,1). During reactivation, HSV-1 virions travel by anterograde transport; that is, from the cell soma of the sensory neuron to the epithelial cells where the primary infection arose (Figure 1A,2). Another pathway for viral spread is the trans-synaptic route, e.g., from one neuron to an adjacent one across the synaptic cleft (Figure 1A,3). Unlike other neurotropic viruses that do not cross synapses, such as Moloney murine leukemia virus (MMLV), lentivirus, adeno-associated virus (AAV), or human adenovirus 5 (Ad5) [34], trans-synaptic spread is a major mode of HSV-1 propagation [35,36]. In fact, given their ability to spread bidirectionally along multi-synaptic pathways, herpesviruses, particularly HSV-1 and PRV, were the first to be widely used to trace neuronal circuits, providing information about connectivity between different areas of the brain [34,37,38,39]. Using HSV-1 as a trans-neuronal tracer, the virus has been observed to cross the synaptic space to label third- to fourth-order neurons [40].

After infection of sensory neurons, HSV-1 travels retrogradely to the cell bodies and establishes a latent infection in the TG (Figure 1B). All branches of the trigeminal nerve (ophthalmic, maxillary, and mandibular) may serve as portals for HSV-1 (Figure 1C), with entry from the oral and nasal epithelia or the cornea. Occasionally, the virus may infect the CNS if it spreads trans-synaptically from the TG to the trigeminal nucleus in the brainstem, from where it might disseminate to higher brain areas (Figure 1B). In fact, there are polysynaptic pathways from the brainstem to the thalamus and somatosensory cortex (Figure 1C) that might be hypothetically utilized by the virus. However, in human HSV-1 infections, viral antigens are mostly found in the olfactory pathways, the temporal cortex, and the limbic system, but not in higher brain areas related to the trigeminal projection pathways, such as the somatosensory cortex [41,42,43,44]. Therefore, those findings support the hypothesis of olfactory spread to the CNS in humans, although spread from the trigeminal nerve to the orbitofrontal and medial temporal lobes has been also proposed. In this regard, the meninges of the middle and anterior fossae are innervated by nerves derived from the TG, and the trigeminal nerve projects to the dura mater via the tentorial nerve, which arises from its ophthalmic division [45]. Spread of the virus to the anterior and middle fossae via tentorial nerves has been proposed to explain viral dissemination to frontal and temporal lobes [46]. However, if the trigeminal pathway was the main portal to the CNS, a higher rate of HSV-1 encephalitis affecting the brainstem would be expected [44]. Finally, the evidence is not sufficient to establish solid conclusions, and further studies are necessary to fully clarify this aspect regarding pathways of HSV-1 entry into the CNS.

Besides the trigeminal nerve, HSV-1 may enter the CNS via the sensory neurons of the olfactory neuroepithelium (Figure 2A). From there, the virus may access the OB and then spread through the olfactory tract to reach limbic structures such as the hippocampus, amygdala, or orbitofrontal cortex (Figure 2A). In the olfactory neuroepithelium, the cilia of the olfactory sensory cells covering the upper nasal cavity provide a portal for viral entry (Figure 2B). The dendrites of these neurons are covered by a thin layer of mucus that protects them. The axons of these first-order olfactory sensory neurons gather in bundles that project through the cribriform plate of the ethmoid bone to reach the OB, forming the olfactory nerves. Once in the OB, the olfactory sensory neurons synapse with mitral and tufted cells in the glomeruli; the virus may infect these cells trans-synaptically at the glomeruli and spread along the olfactory tract towards the olfactory projection pathways. In the olfactory tract, HSV-1 may access the ipsilateral olfactory projection areas via the lateral olfactory stria. However, virus circulating along the medial olfactory stria may reach the contralateral olfactory structures via the anterior commissure (Figure 2C).

### 3.1. HSV-1 Receptors in the CNS

HSV-1 may enter cells by membrane fusion at the cell surface or by endocytosis [47,48,49]. Four glycoproteins (gB, gD, gH, and gL) are required for viral entry, and gC is involved in cell attachment, although it is not essential to enter cells [50,51]. Heparan sulphate proteoglycans (HSPGs) act as attachment receptors and gD interacts with nectin-1, herpes virus entry mediator (HVEM), and 3-O-sulphated heparan sulphate to enter cells. Cell receptors for gB are the paired immunoglobulin-like type 2 receptor alpha (PILRα), the myelin-associated glycoprotein (MAG), and the non-muscle myosin heavy chain IIA (NMHC-IIA) [47].

Heparan sulphate (HS) is a linear, negatively charged glycosaminoglycan (GAG) consisting of N-acetylglucosamine and glucuronic acid residues that may be O- or N-sulphated at different positions. This polysaccharide is highly expressed as HSPG on the surface and extracellular matrix of most animal cell types [52,53]. Since most human herpesviruses use them as cell attachment factors, the polarized distribution of HSPGs may modulate viral entry [54], although the distribution of entry receptors may also play a crucial role in influencing the polarity of infection. Nectin-1 has been observed abundantly beneath the HS-positive cilia of the sensory neurons in the olfactory neuroepithelium [18]. In most epithelia, HSPGs are mainly expressed in the basolateral domain [55]. However, in the olfactory epithelium, HSPGs and nectin-1 are also localized to the apical domain, thus facilitating viral binding and providing an important herpesvirus entry portal [56].

HSV-1 entry into neurons takes place by fusion between the viral envelope and the plasma membrane of the sensory neuron dendrites. Therefore, unlike other neurotropic viruses—such as rabies or polioviruses, which enter cells by endocytosis—HSV-1 and alphaherpesviruses in general deliver naked capsids into the host cytoplasm [57]. Then, nucleocapsids travel towards the microtubule organizing center (MTOC) by dynein-mediated retrograde transport and eventually reach the nucleus to implement viral replication. The tegument plays a crucial role in this process, since it participates in the recruitment of the dynein–dynactin complex [57]. To exit neurons and infect adjacent epithelial cells or higher-order neurons, HSV-1 travels along the axons by microtubule-directed anterograde transport. Two hypotheses, the so-called “separate” and “married” models, have been proposed for this transport. In the “separate” model, naked viral nucleocapsids lacking viral envelope and membrane glycoproteins are transported in axons, whereas in the “married” model, enveloped particles enclosed in vesicles are transported in the anterograde direction. It is not clear which process is used by HSV-1 to exit neurons, and it has been even proposed that both mechanisms may be used [58,59].

Nectin-1 expression has been reported in the limbic system, frontal association cortex, and OB in adult mice [60]. In fact, the distribution of this entry receptor in the brain undergoes spatiotemporal changes throughout development. Thus, in the newborn murine brain, expression of nectin-1 was mostly observed in the cerebral cortex, corpus callosum, and anterior commissure, whereas on the seventh postnatal day, this expression increased in limbic regions and decreased in association areas [60]. This developmental change in the distribution of viral receptors may explain the change in susceptibility to HSV-1 infection during brain development. In fact, HSV-1 encephalitis in adults typically affects the orbitofrontal and medial temporal lobes and the insula, whereas neonatal encephalitis shows a much more diffuse inflammation and necrosis [44,46]. Other studies in murine models have reported that nectin-1 mediates infection of dorsal root ganglia neurons [61] and the cornea [62].

In the human brain, nectin-1 has been has been detected in the cornea, neocortex, and hippocampus of formalin-fixed paraffin-embedded tissues from autopsies [63]. Expression of nectin-1 has been also shown to be necessary for CNS infection with HSV-2 and for triggering encephalitis [64]. Later reports confirmed that expression of both gB and gD receptors, specifically nectin-1, HVEM, NMHC-IIA, and possibly MAG, is higher in the hippocampus, suggesting that differential expression levels of these viral receptors in the adult hippocampus might be responsible for the susceptibility of this area to HSV-1 infection [65].

### 3.2. HSV-1 Entry Pathways

In vivo, herpesviruses have demonstrated tropism for the olfactory epithelium, but not the respiratory epithelium [5]. Thus, intranasal infection of mice with HSV-1 mostly targeted the olfactory neuroepithelium, although the respiratory epithelium was affected to some extent. From this site, the virus travelled to the TGs and then returned peripherally without causing significant neurological disease, nor spreading to the OB [18]. However, other studies in murine models have found HSV-1 in the OBs and higher brain regions after intranasal inoculation [40,66]. The brainstem is another main target of HSV-1 infection; after intranasal inoculation of mice with HSV-1, viral antigens were detected not only in neurons of the TG, but also in trigeminal nuclei and reticular formation, including the raphe nuclei and locus ceruleus [67]. In another study with intranasally infected mice, HSV-1 was detected in the trigeminal root entry zone in the brainstem and the OBs. Later, the virus spread to other brainstem nuclei, and in some mice to the thalamus and cerebellum. From the OBs, virus spread to olfactory projection areas such as the anterior olfactory nucleus, lateral olfactory tract, temporal lobe, or hippocampus [68]. On the other hand, following intracorneal inoculation, the virus was also detected in the OBs, possibly as a result of the passage of the virus to the olfactory neuroepithelium along the nasolacrimal duct [68]. In addition, direct inoculation of HSV-1 into the OB of rabbits led to an infection localized to the frontal and temporal cortices of the animals [69].

Another approach in animal models is the use of corneal inoculation to infect the ophthalmic branch of the trigeminal nerve. Using this route, the infection extends along the trigeminal pathways [70], olfactory system, and limbic structures, such as the entorhinal cortex [71]. Studies of HSV-1 spread using corneal infection of mice have also contributed significant information about viral dissemination. Early studies showed that corneal inoculation of HSV-1 triggers productive infection, not only in peripheral ganglia, but also in brain tissue, indicating viral spread from the PNS to the CNS [72]. More recent studies revealed that HSV-1 may spread to the OB as early as the trigeminal nerve following ocular infection, later inducing a chronic inflammatory response in the OB during latency [73]. Regarding mandibular division, inoculation of the virus into this branch of the trigeminal nerve produced encephalitis, affecting the temporal cortex and limbic system [74]. In addition to the olfactory neurons, in animals HSV-1 may also enter the CNS via the sensory neurons of the vomeronasal organ [75]. From here, the infection is transmitted to the accessory OB and then to the amygdala and hypothalamus [75].

It has also been demonstrated that the anterior commissure may serve as a pathway for contralateral spread of HSV-1. After nasal inoculation of rats, HSV-1 initially infected the right TG and the right OB, and then spread to the same structures on the contralateral side. That spread to the left hemisphere was shown to proceed via the anterior commissure [76]. In addition, that study suggested that HSV-1 used two pathways to spread in the brain. In the first one, the virus was internalized by neurons in the olfactory mucosa and transported to the mitral cells within the right OB. The virus then spread via the right olfactory tract and crossed the anterior commissure to finally reach the left olfactory tract, from where it travelled towards the left OB. The second route consisted of infection entering the right TG and infecting the medulla oblongata and the cerebellum. The hippocampus was infected either from the olfactory tract or the TG. The aquaporin 9 (AQP)-positive cells infected with HSV-1 in the anterior commissure were proposed as OLs, since staining of infected cells of the anterior commissure were negative for markers of astrocytes, microglia, or neurons, and the AQP9-positive cells morphologically resembled OLs. The virus might exploit these myelinating cells to spread rapidly, since a single OL may myelinate several axons [76].

In humans, the olfactory route seems to be highly relevant in symptomatic and asymptomatic HSV-1 infections; additionally, HSV-1 encephalitis in adults typically affects the limbic system. Immunostaining of HSV-1 in autopsied encephalitis cases revealed the presence of viral antigens in the temporal lobe, hippocampus, amygdala, olfactory cortex, insula, and cingulate gyrus. The virus was also found in glial cells of the olfactory tract [77]. Paradoxically, the entry of HSV-1 to the CNS via the trigeminal nerve is unclear, although the sensory neurons of the TG are the major site of latency [5].

### 3.3. HSV-1 Reactivation

Rabbits and mice have traditionally been the major animal models used to study HSV-1 infection, reactivation, and spread to the CNS [78]. To detect latent HSV-1 in the peripheral sensory TG or dorsal root ganglia, early approaches used co-cultivation of explants with permissive cells [19,79]. During latency, infectious virus cannot be detected after inoculating susceptible cells with ganglion homogenates, but it is possible after incubation of susceptible cells with intact ganglionic explants [19]. In contrast, infectious virus was rarely recovered from the CNS of latently infected animals after explant culture [80], although latent states can be detected in the sensory ganglia or CNS using PCR-based techniques [81].

In humans, several systemic factors such as emotional stress or UV light exposure have been implicated in HSV-1 reactivation. In experimental animal models, this process has been induced by different methods, such as axotomy, cold restrain stress, hyperthermia, hypothermia, corneal scarification, or immunosuppression [82,83]. It has also been demonstrated that epinephrine may induce HSV-1 reactivation in animal models [84,85,86], consistent with the role of stress in HSV-1 reactivation [87,88,89,90]. Iontophoresis is a process that uses an electrical current to transport ions or charged molecules into a tissue. Using this method, epinephrine can be directly conveyed into rabbit eyes to ensure its penetration into ocular tissues of the whole cornea without any trauma [91]. In an early study with the rabbit model, latently infected animals, after prior infection via the cornea, were submitted to reactivation using iontophoresis of epinephrine. Infectious HSV-1 was recovered from the superior cervical ganglion, the TG, and the ophthalmic branch of the trigeminal nerve, suggesting that in addition to TG, the superior cervical ganglion may also harbor HSV-1 during latency [92].

It was initially supposed that reactivation occurred in the sensory ganglia and that brain infection was originated by the spread of reactivated virus from those ganglia. However, later findings have cast doubt on that hypothesis. In a latent-infected mouse model, hyperthermic stress caused reactivation of HSV-1 in the brain earlier than in the TGs [93]. That study, which showed CNS reactivation in explants, suggested that recurrent brain infection might be prompted by the latent virus within the brain itself, not by the spread of the reactivated virus from the ganglia. Nevertheless, a more recent report had conflicting results—a study of reactivation in the TGs and the brainstem detected HSV-1 from both the TG and brainstem of latently infected mice following a reactivation stimulus, but a higher frequency of reactivation and increased infectious titer were recovered from the TGs [94]. Since both studies were developed with C57BL/6 mice, excluding the model as a source of discrepancy, the contradictory results are possibly due to differences in methodology. To directly compare the frequency of reactivation and the amount of infectious virus produced in the TG and brainstem after hyperthermia, Doll et al. used a quantitative assay in which recovery of infectious virus was related to tissue weight. In addition, they used a direct method to detect HSV reactivation in the brainstem, whereas Yao et al. used a two-step assay, which may imply a high contamination risk [94]. Further research will be necessary to fully clarify these conflicting results and to definitively determine the dynamics of HSV-1 reactivation in the nervous system.

## 4. HSV-1 and Demyelination

MS is an immune-mediated demyelinating and neurodegenerative disease of the CNS of unknown etiology, although several viruses are known to be involved in such demyelinating diseases [6,8,95,96]. The disease is multifactorial, influenced by genetic and environmental factors [97], and it is characterized by multifocal demyelinating lesions in both the white and gray matter [98] of the brain and spinal cord. These lesions can be associated with axon degeneration and synaptic loss. MS is typically multifocal and multiphasic (relapsing), and lesions are thought to be caused by infiltration of immune cells into the CNS [99].

One hallmark of MS is the presence of oligoclonal IgG bands (OCBs) in the cerebrospinal fluid (CSF) of patients. These OCBs, which indicate an anomalous intrathecal B-cell response, are found in the CSF of more than 95% of MS patients and cannot be detected in serum. OCBs are typically detected in inflammatory and infectious CNS disorders. In addition to MS, there are other pathologies with reported CSF OCBs, such as systemic lupus erythematosus, aseptic meningitis, HIV infection, and HSV-1 encephalitis [99]. It has been suggested that OCBs are directed against the infectious agent that causes the disease, and that MS might be triggered by an agent against which the antibody response in the brain and CSF was directed [100]. In fact, OCBs from patients with infectious CNS diseases have been proven to recognize the relevant infectious agent [101]. Regarding MS, OCBs directed against EBV and HHV-6 have been identified in patients [102]. OCBs directed against HSV-1 in the CSF of patients with MS has also been reported [103], although other studies did not find reactivity to HSV-1 antigens [104]. Regardless, the antibody activity of most OCBs remains unknown to date.

It is known that human oligodendrocytic cells are susceptible to HSV-1 in vitro [105]. In murine models, infected OLs were found in the mandibular division of the spinal trigeminal tract after infection of mice through cranial nerve XII [106]. Early studies with animal models showed latent TG infections and demyelinating lesions in mice intranasally infected with HSV-1 [107]. Later research also reported that mice infected with HSV-1 can develop lethal encephalitis or virus-induced CNS multifocal demyelinating lesions, with outcome affected by several factors, including the route of infection and mouse strain [108,109,110,111,112]. Demyelinating lesions have also been associated with HSV-1-induced facial nerve paralysis [113]. A recent study demonstrated a direct association between infection with HSV-1 and multifocal brain demyelination in a murine model [114]. Moreover, in that study, demyelination was followed by remyelination, although it was incomplete and the presence of scars was observed. As in studies with experimental animals, resistance to HSV-1 varies between primary cultures of human OLs and is donor-dependent [115]. Susceptibility to HSV-1 encephalitis may be caused at least partly by mutations in Toll-like receptors that decrease the intrinsic resistance of CNS cells (neurons and OLs in particular) to HSV-1 infection [116,117].

An association between HSV-1 infection and demyelination has been also suggested from studies with human patients. HSV was detected early in the CNS of an MS patient [118], and later HSV-1 was also isolated from the CSF of a patient during the first attack of MS [119]. Postmortem brain samples from 37 cases of MS were screened for HSV-1 and HSV-2, finding higher prevalence of HSV in MS patients compared to controls and in more active plaques than inactive plaques [120,121]. A case of acute MS preceded by varicella-zoster virus (VZV) infection was reported at the same time as intrathecal reactivation of HSV-1 and HHV-6 [122], and a coincident onset of HSV-1 encephalitis and MS has been also described [123]. In another study, patients of MS treated with valacyclovir showed a reduction in the number of new active demyelinating lesions and a decrease in the number of scans free of new active lesions [124]. HSV-1 may also play a role in triggering MS relapses during clinical acute attacks of MS, at least in the most frequent clinical presentation of the disease, the relapsing–remitting form [125]. HSV-1 DNA was detected by PCR in peripheral blood mononuclear cell (PBMC) samples from relapsing–remitting MS patients [126]. However, the involvement of HSV-1 in MS etiology is far from confirmed, and other investigators have proposed other herpesviruses as more plausible etiological agents [127,128,129], or even doubt the role of herpesviruses in the etiology of demyelinating diseases [130].

Viruses, specifically HSV-1, might not operate as unique causative agents, but rather as risk factors, and indeed genetic susceptibility and the immune system are also crucial in demyelinating pathologies. For instance, when a recombinant HSV-1 constitutively expressing interleukin-2 (IL-2) was inoculated into mice, it provoked CNS demyelination and optic neuropathy, whereas infection with recombinant viruses expressing IL-4, gamma interferon, IL-12p35, IL-12p40, or IL-12p70 did not induce this effect [131]. On the other hand, donor-dependent differences in resistance to infection with HSV-1 were established in primary cultures of human OLs [115].

### 4.1. HSV-1 and Endogenous Retroviruses

Endogenous retroviruses (ERVs) are vestiges of ancient retroviral infections that remain in the host genomes of all vertebrates. They make up around 8% of the human genome and their expression may be triggered by environmental factors, inducing pathogenesis under some circumstances. Several human ERV (HERV) transcripts and proteins have been identified in the CNS, often associated with neuroinflammation [132], and there is a solid epidemiological association between MS and the expression of ERVs, which are upregulated in the brains of MS patients compared to healthy controls [12,133,134,135,136]. The MS-associated retrovirus (MSRV), a member of the HERV-W family, has been frequently isolated from MS patients [137], and its presence in the CSF of these patients has been associated with a greater rate of disability and progression of the disease [138]. Herpesviruses have been implicated in regulation of the HERV-W family [139], and a role for HSV-1 in HERV-W [140] and HERV-K [141] expression has been reported. HERV-W–MSRV expression may be enhanced by HSV-1 in leptomeningeal cells [142,143]. HSV-1 can activate HERV-W in cells involved in MS pathogenesis, such as B cells, macrophages, microglia, and astrocytes [144], and may induce ERV proteins [145].

Syncytins are Env glycoproteins encoded by ERV genes that are involved in mammalian placental morphogenesis [146,147]. These proteins stimulate cell–to–cell fusion in a process analogous to viral entry, promoting the formation of syncytia [146], and can activate pro-inflammatory and autoimmune processes [148]. Syncytin-1, an Env glycoprotein encoded by the HERV-W env gene, plays a crucial role in placental trophoblastic formation and has an immunosuppressive role that impedes rejection of the fetus by the maternal immune system. However, this protein has also been associated with different pathogenic processes, triggering neuroimmune activation and OL damage [148]. In this regard, syncytin-1 may inhibit the differentiation of oligodendroglial precursors, thus hindering remyelination [149]. In astrocytes, overexpression of syncytin-1 (which is upregulated in glial cells in demyelinating lesions of MS patients) triggered the release of redox reactants, inducing neuroinflammation and death of OLs [150]. In this context, HSV-1 has been demonstrated to upregulate syncytin-1 [145], and therefore deepening the understanding of this role may greatly increase knowledge of the demyelinating processes.

### 4.2. HSV-1 and Molecular Mimicry

Another mechanism that has been associated with HSV-1-related demyelination is molecular mimicry. In this process, the peptides of a pathogen are similar to those of the host organism, triggering activation of autoreactive immune cells in susceptible individuals. Viruses may induce autoimmunity by molecular mimicry [151,152], and several viral peptides have been shown to activate autoreactive T cells [153]. The triggering of autoimmunity by HSV-1 infection was demonstrated when an epitope expressed by the capsid protein was recognized by autoreactive T cells targeting corneal antigens in a murine model of autoimmune herpes stromal keratitis [154]. Mimicry between an epitope shared by the HSV-1 glycoprotein gB and a brain-specific factor has also been reported, supporting the hypothesis that viral infections may prompt the production of self-reactive CSF antibodies [155].

After a HSE episode, a complex immune cellular and humoral response starts. Cytotoxic T lymphocytes lyse cells infected by HSV-1 and the production of inflammatory cytokines (predominantly by Toll-like receptor [TLR] 2) commences [156]. The presence of cytokines such as IL-6, interferon gamma, or TNF alpha, which are detected in the serum and CSF of HSE patients, indicates a strong immune response and suggests that inflammation may contribute to the pathological impact of the viral infection [156]. However, autoimmunity may be another effect triggered by infection. In fact, HSV-1 infection may induce antibodies against neurotransmitter receptors. After an episode of encephalitis caused by HSV-1, around 10–20% of patients may experience a relapse syndrome known as post–herpes simplex encephalitis (PHSE) [157]. This syndrome is immune-mediated, and many patients acquire antibodies against the ionotropic glutamate NMDA receptor (NMDAR). In addition to NMDAR, PHSE may trigger the release of antibodies against GABA A and AMPA receptors, or other still unidentified antigens [157,158,159]. However, although a link between HSV-1 and anti-NMDAR encephalitis has been found, it is not completely clear whether or not molecular mimicry is the responsible mechanism [160]. Regarding ocular HSV-1 infection, it has been proposed that virus infection might result in unmasking of corneal autoantigens, leading to chronic autoreactive T cell stimulation. Alternatively, autoimmunity might be explained by a process of molecular mimicry triggered by a viral peptide sharing reactivity to the unmasked corneal autoantigen. Thus, the initial antiviral response against the virus might subsequently become sustained by autoreactive T_aggressor_ cells [157].

### 4.3. HSV-1: A Role in Remyelination Impairment?

Primary demyelination in the CNS is a process by which a direct injury to OLs harms the myelin sheath. On the contrary, in secondary demyelination (Wallerian degeneration), the myelin sheath degenerates as a result of primary axonal loss [161]. After demyelinating processes, a mechanism of remyelination starts, which aims to repair the damaged myelin. This process is critically regulated by numerous intracellular signaling pathways [162,163] and depends on generation of new mature OLs derived from a population of adult CNS precursor cells (adult OL precursor or progenitor cells (OPCs)). Remyelinating OLs can derive from the adult subventricular zone (SVZ), a region which may be a source of remyelinating OLs during MS, although the contribution of cells derived from that region might be small compared to the local sources [161].

It has been suggested that infection of OPCs with HHV-6 might impair remyelination [164]. In response to demyelinating damage, OPCs proliferate and migrate to the lesion site, where they differentiate into myelinating OLs and wrap damaged axons with new myelin sheaths. A hypothetical viral infection of OPCs leading to impairment of differentiation or migration might affect remyelination in patients with demyelinating diseases [164]. In this context, HSV-1 might exert a similar role, as it has been proven to infect OPCs in vitro, although the infection increases along with differentiation [165]. Therefore, infection of a population of OPCs by HSV-1 during remyelinating processes might affect this process, resulting in impairment of remyelination.

## 5. Conclusions

Several viruses are involved in demyelinating diseases such as MS, an immune-mediated demyelinating disease of the CNS of unknown etiology. This disorder is influenced by genetic and environmental factors. Among them, a viral trigger or risk factor has been considered possible. OLs, the myelin-forming cells of the CNS, are highly susceptible to HSV-1 infection in vitro. In animal models, this virus may induce CNS multifocal demyelinating lesions, while studies with human patients have suggested an association between HSV-1 infection and demyelination. However, HSV-1 may act as a risk factor for MS progression rather than as a causative agent. Processes such as molecular mimicry, regulation of ERVs, or remyelination might be mediated by this neurotropic pathogen.
